# Identification of high-risk factors as indicators for adjuvant therapy in stage II colon cancer patients treated at a single institution

**DOI:** 10.3892/ol.2013.1433

**Published:** 2013-07-01

**Authors:** KEIZO YAMAGUCHI, YUTAKA OGATA, YOSHITO AKAGI, KAZUO SHIROUZU

**Affiliations:** Department of Surgery, Kurume University School of Medicine, Kurume, Fukuoka 830-0011, Japan

**Keywords:** adjuvant chemotherapy, bowel obstruction, carcinoembryonic antigen, stage II colon cancer, colorectal cancer

## Abstract

Although post-operative adjuvant chemotherapy (ACT) is only recommended for patients with stage II colon cancer who are at a high risk of recurrence, the definition of high risk remains unclear. The present study aimed to identify the risk factors for recurrence, which may also be indicators for adjuvant therapy, using a retrospective analysis of clinicopathological data obtained from stage II colon cancer patients who had undergone a curative resection. The present study also investigated the effects of ACT in patients who displayed the risk factors for recurrence. Univariate and multivariate analyses of the data collected from 377 stage II colon cancer patients, treated at Kurume University Hospital (Fukuoka, Japan) between 1982 and 2005, was conducted in order to determine and compare the risk factors for recurrence between the 163 patients who had undergone adjuvant therapy and the 214 patients who had not undergone adjuvant therapy. The risk factors for recurrence in patients who had not undergone adjuvant therapy were a serum carcinoembryonic antigen (CEA) level that was twice the cut-off value and pre-operative bowel obstruction. Adjuvant therapy provided no benefit to patients who presented with neither risk factor, but significantly decreased the recurrence rate in patients presenting with one or both risk factors. Based on these findings, serum CEA levels of twice the cut-off value and pre-operative bowel obstruction were proposed as indicators in the assessment for adjuvant chemotherapy following a curative resection for stage II colon cancer. These results warrant further clinical study of ACT in patients with one or both risk factors.

## Introduction

The current guidelines to treat stage II colon cancer patients include the recommendation that post-operative adjuvant chemotherapy (ACT) should only be considered for patients who have a high risk of recurrence (1,2). Although the definition of high risk has not been completely established to date, various risk factors have been proposed. The current American Society of Clinical Oncology (ASCO) Guidelines recommend the provision of ACT for patients with inadequately-sampled nodes, T4 lesions, tumor perforation or poorly-differentiated histology (3). The current European Society for Medical Oncology (ESMO) Clinical Practice Guidelines define patients with stage II colon cancer to be at high risk and a candidate for adjuvant therapy if at least one of the following characteristics are identified: Lymph nodes sampling <12; a poorly-differentiated tumor; vascular, lymphatic or perineural invasion; tumor presentation with obstruction or tumor perforation; and pT4 stage (4).

In accordance with the ASCO and ESMO guidelines, the current Japanese guidelines for the treatment of colorectal cancer recommend adjuvant therapy for patients at a high risk of recurrence (5). Based on extensive research, the Japanese Study Group for Post-operative Follow-up of Colorectal Cancer, a multicenter collaborative study group, recently recommended ACT for patients who meet two or more of the following criteria: Extensive venous invasion; <13 dissected lymph nodes; an age of >50 years; and/or being of the male gender (6). In a previous examination of stage II colon cancer patients, it was identified that the patients who had presented with invasive gross tumors and elevated pre-operative serum carcinoembryonic antigen (CEA) levels and who had not undergone ACT were at a high risk of recurrence (7). Accordingly, in the present study, the patients with elevated pre-operative serum CEA levels were identified as candidates for ACT. The majority of studies conducted prior to the present study were based on a comparison of data that was collected from patients who had or had not undergone ACT. As a result, the effect of chemotherapy was not excluded as a confounding factor in the analysis of risk factors for recurrence. In addition, the effect of age or gender, which may not be associated with the malignant potential of colon cancer, cannot be excluded in the analysis of prognostic factors in terms of overall survival (OS), commonly used in past studies. To address these limitations, the time to recurrence (TTR), a relatively objective measure, was used to determine the prognosis of the stage II colon cancer patients who had not undergone ACT following a successful curative resection, and to compare the prognosis with that of patients who had undergone adjuvant therapy in order to identify the risk factors for recurrence that may be indicators for adjuvant therapy (8).

## Materials and methods

### Patients and specimens

The present retrospective study examined data collected from 377 patients who were treated at a single medical institution (Kurume University Hospital, Kurume, Fukuoka, Japan) between 1982 and 2005 for stage II colon cancer. All patients met the study criteria of i) having been pathologically diagnosed with stage II colon cancer, ii) having undergone a curative resection with lymphadenectomy and iii) not having undergone pre-operative chemotherapy, radiotherapy or immunotherapy. Of these 377 patients, 163 had undergone ACT and thus constituted the ACT group and 214 patients had not undergone ACT and thus constituted the surgery alone (SA) group. The primary reasons as to why ACT had not been administered were refusal to provide informed consent and the patient age being ≥75 years. Approval for the study was obtained from the Kurume University Hospital Ethics Committee.

### Diagnosis and staging procedures

The pathological factors and stage classification of colon cancer were determined according to the TNM classification system developed by the International Union against Cancer (UICC) (9). Mesenteric lymph nodes had been removed from the mesenteric adipose tissue for histological examination immediately after surgery. A pathological examination of all isolated lymph nodes was performed, with the histopathological examination being performed using a 5-mm thick longitudinal whole tissue section. Lymphatic permeation and venous invasion was determined on the basis of previously defined criteria (10,11).

### ACT

Oral fluoropyrimidines, including tegafur plus uracil (UFT; 300 mg/day per 1 m^2^ body area) and doxifluridine (5′-DFUR; 600 mg/day per 1 m^2^ body area), an intermediate metabolite of capecitabine, were the agents that were administered during a course of post-operative ACT that was provided for >6 months.

### Follow-up schedule and examinations

All patients were monitored as outpatients according to a regular examination schedule. The final follow-up date for the present study was April 30, 2011. The post-operative surveillance consisted of measuring the tumor marker levels, chest radiography and abdominal ultrasonography, in addition to a physical examination every 3 to 6 months for the first 3 years, every 6 months for the next 4 years and annually thereafter. Chest and abdominal computed tomography or magnetic resonance imaging was performed every 6 to 12 months for the first 3 years and then annually or when recurrence was suspected thereafter.

### Statistical analysis

The TTR was calculated using the day of the first surgery as the start date and considering recurrence of the primary cancer or mortality due to the primary cancer as an event. TTR curves were then generated using the Kaplan-Meier method and the significance between groups was determined using the log-rank test. Univariate and multivariate analyses of the clinicopathological factors, including the CEA level associated with the TTR, were performed using Cox’s proportional hazards model. The analysis of the differences in the clinicopathological factors between the groups was performed using Fisher’s exact test and Student’s t-test. All statistical analyses were performed using JMP version 9.0.2 software (SAS Institute, Inc., Cary, NC, USA) and P<0.05 was considered to indicate a statistically significant difference.

## Results

### Patient clinicopathological data

The clinicopathological data of the patients are presented in Table I. The tumors were located in the left colon (descending, sigmoid or rectosigmoid colon) in 225 patients and the right colon (cecum, ascending colon or transverse colon) in 152 patients. The classification of the tumor according to the gross tumor type was invasive in 39 patients and non-invasive in 338 patients. An increased pre-operative CEA level was observed in 136 patients (36.1%), 25 (18.4%) of who later experienced recurrence. In addition, a CEA level of twice the cut-off value was observed in 68 patients (18.0%), 15 (22.1%) of whom later experienced recurrence (Table II). The tumor diameter was greater than the median tumor diameter of 55 mm in 203 patients.

Among the total patients, there were 337 in whom ≥12 lymph nodes had been sampled and the histological examination indicated that the tumor was well-differentiated in 276 patients, moderately-differentiated in 64 and poorly-differentiated/other in 37. A total of 25 patients were diagnosed with a pre-operative bowel obstruction; these patients were defined as those who required the placement of an oral or transanal decompression tube or had undergone emergency surgery for pre-operative bowel obstruction. Perforations had been observed in two of these patients, who were therefore included in the group of patients with bowel obstructions. Extensive lymphatic permeation was observed in 45 patients and extensive venous invasion in 33 patients.

The overall median follow-up duration was 98 months. Recurrence was observed in 48 patients (12.7%), of whom 14 (8.6%) were in the ACT group and 34 (15.9%) were in the SA group. A statistical analysis of the results indicated a significant difference between the two groups in terms of recurrence (P=0.042; Table II), age (P=0.001), number of dissected lymph nodes if <12 nodes had been sampled (P=0.006) and the extent of venous invasion (P=0.026; Table I).

### Univariate and multivariate analyses

The results of the univariate analysis, which used Cox’s proportional hazards model to examine the TTR of the SA group, revealed that the CEA levels were twice the cut-off value and that bowel obstruction and extensive lymphatic permeation were significant risk factors for recurrence (Table III). The statistical analysis of the significance of a CEA level above the cut-off value, twice the cut-off value and three times the cut-off value indicated that a level that was twice the cut-off value had the highest hazard ratio (HR) and the least significant association. Therefore, a CEA level of twice the cut-off value was used in the subsequent analyses. The multivariate analysis of the parameters that were identified as significant in the univariate analysis confirmed that a CEA level of twice the cut-off value and bowel obstruction were significant risk factors for recurrence.

### Effect of ACT on patients with risk factors for recurrence

Subsequent investigation of the effect of post-operative ACT in patients with the previously mentioned risk factors for recurrence showed no significant differences in the recurrence rate in patients with and without a CEA level of twice the cut-off value in the ACT group (P=0.999). However, the recurrence rate was significantly higher in patients with a CEA level of twice the cut-off value in the SA group (P=0.003). In addition, the rate of recurrence in patients in the ACT group who had presented with a CEA level of twice the cut-off value was identified to be significantly lower than that of patients in the SA group (P=0.008; Table IV).

No significant differences were identified in the rate of recurrence between patients in the ACT group who were and who were not diagnosed with a pre-operative bowel obstruction (P=0.999). In contrast, within the SA group, the rate of recurrence was shown to be significantly higher in patients who were diagnosed with pre-operative bowel obstructions than in those who were not (P=0.018; Table V). A comparison of the patients in the ACT and SA groups who were diagnosed with a pre-operative bowel obstruction revealed that the rate of recurrence of the patients in the ACT group was lower than that of the patients in the SA group (P=0.051; Table V).

**Figure 1:**
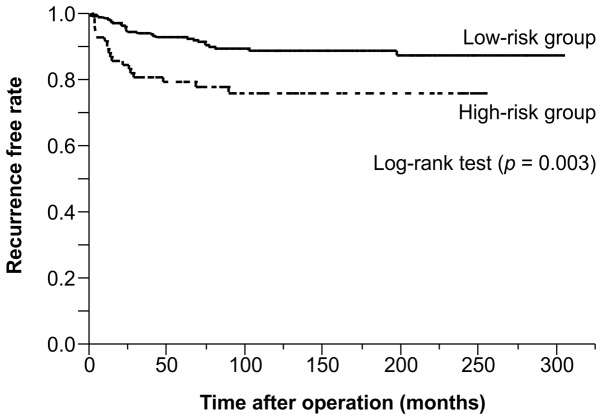
Time to recurrence (TTR) curves of all patients by risk group.

**Figure 2:**
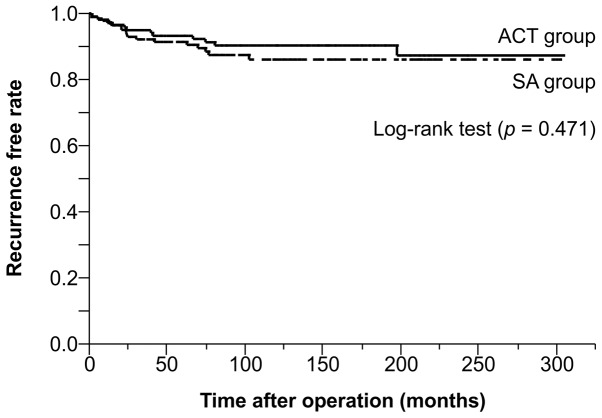
Time to recurrence (TTR) curves of low-risk patients. ACT, adjuvant chemotherapy; SA, surgery alone.

**Figure 3:**
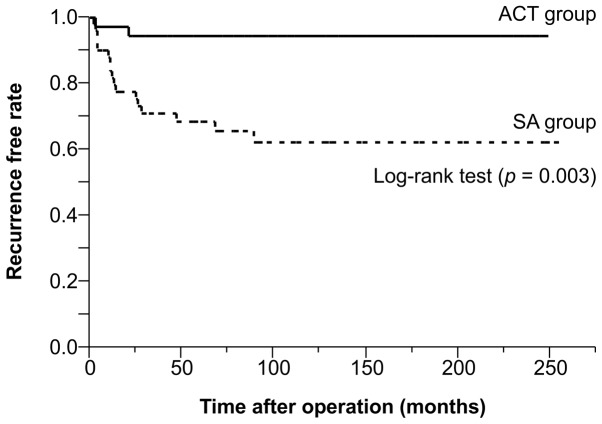
Time to recurrence (TTR) curves of high-risk patients. ACT, adjuvant chemotherapy; SA, surgery alone.

A multivariate analysis confirmed that a CEA level of twice the cut-off value and a diagnosis of a pre-operative bowel obstruction were significant factors in predicting the rate of recurrence. Based on these results, the 377 patients were divided into two groups. Those who had presented with at least one of the risk factors (CEA level of twice the cut-off value and/or a diagnosis of a pre-operative bowel obstruction) were assigned to the high-risk group (n=86) and those who had presented with neither risk factor were assigned to the low-risk group (n=291). A between-group comparison, which calculated the TTR using the Kaplan-Meier method, revealed a significantly lower rate of recurrence in the low-risk group (Fig. 1). A within-group analysis of the low-risk group indicated no significant differences in the rate of recurrence between the low-risk patients in the ACT and SA groups (Fig. 2). However, the within-group analysis indicated a significantly lower risk in the high-risk patients in the ACT group than in the high-risk patients in the SA group (Fig. 3).

## Discussion

Patients with stage II colon cancer who are only treated with surgery are generally considered to have a better prognosis if a curative resection is possible. Previous studies have estimated the recurrence and 5-year OS rates following the treatment of stage II colon cancer to be 7.9–22 and 75–92.5%, respectively, and have shown that ~20% of patients experience recurrence within 5 years (1,12,13). In Japan, the recurrence and 5-year OS rates are estimated to be 13.3–13.8 and 83.7%, respectively (6,14). While these rates are comparable with the 12.7 and 82.6% rates identified for recurrence and OS, respectively, in the present study, when all the patients were analyzed, the rates did not reflect the significant differences in the recurrence and 5-year OS rates between patients in the ACT group (8.6% and 91.0%) and the SA group (15.9% and 76.1%; P<0.0001). Although the improved prognosis of the ACT group may have been due to selection bias, identifying the high-risk factors for recurrence is fundamental for determining the form of adjuvant therapy that is most likely to be effective.

Among the various endpoints that may be used in a study of adjuvant therapy, Punt *et al* recommended the use of the disease-free survival (DFS) rate as the primary endpoint and the TTR as the secondary endpoint (8). However, as the present study aimed to identify the high-risk factors for recurrence, the TTR was used as the endpoint instead of DFS or OS rates to exclude the effect of mortality due to other causes or other cancers. With regard to the correlation between prognosis and gender, previous studies have revealed that while there are no significant differences in OS or disease-specific survival (DSS) rates between male and female colon cancer patients (15,16), male colon cancer patients have a poorer prognosis (6,17–19), experience a significantly higher rate of post-operative mortality and are more likely to succumb to adverse cardiovascular events (17). These findings indicate that colon tumors have the same biological malignant potential in male and female patients, but that males are more vulnerable to surgical stress (16). Nevertheless, female colorectal cancer patients have also been identified to have a poor prognosis (19), and thus a consensus remains to be achieved with regard to the correlation between gender and prognosis. With regard to the correlation between prognosis and age, elderly patients have been identified to have low rates of survival, which has been attributed to the consideration of all mortalities as events and the low rate of administration of adjuvant therapy to this population (16). However, such attribution is controversial, as a poor prognosis has also been reported in younger patients (20).

In the case of all events, gender and age are highly likely to affect the inherent biological behavior of the patient, rather than any difference in the biological malignant potential of the tumor. It is thus unclear whether gender and age should be considered high-risk factors, simply as statistically significant differences have been observed between males and females and among various age groups. Based on this reasoning, the TTR was considered a more acceptable measure in the present analysis of the risk of recurrence than the OS or DFS rates, for which gender or age may have been a confounding risk factor.

In a previous study on stage II colon cancer patients, the invasive gross tumor type, elevated pre-operative serum CEA level and lack of adjuvant therapy were identified as risk factors for a shorter relapse-free survival (7). However, as the patients in the ACT group had shown a better prognosis than those in the SA group, regardless of whether the patients presented with a normal or elevated pre-operative CEA level, the CEA level was considered to be a significant factor in the assessment of indication for post-operative ACT. In contrast, a CEA level of twice the cut-off value and a diagnosis of pre-operative bowel obstruction were identified as independent factors for a shorter TTR in the present study.

Previous studies have identified several pathological factors, including gross tumor type (6,7), depth of invasion (21–23), tumor histology (23) and vascular invasion (6,21,23), and surgical factors, including the number of dissected lymph nodes (6,22), as high-risk factors in stage II colon cancer. Researchers generally interpret the results that are associated with the risk factors that are assessed objectively, including age and gender, in a similar manner. However, the results with regard to the pathological risk factors that are assessed subjectively, including tumor histology and vascular invasion, as well as those regarding the gross tumor type and number of dissected lymph nodes, may be interpreted in a different manner. To avoid inconsistent interpretations of the present results, a serum CEA level of twice the cut-off value and a diagnosis of pre-operative bowel obstruction, which are indicators that are relatively objective and reflective of a high risk prior to surgery, were used in the analysis of the TTR in the present study.

Several studies have also considered pre-operative bowel obstruction to be a risk factor for recurrence in stage II colon cancer patients (23–25). However, as these studies often used various definitions of pre-operative bowel obstruction, leading to wide variations in the rate of obstruction of between 9.8 and 47% (23–26), it is difficult to assess the utility of this symptom as a risk factor. Pre-operative bowel obstruction is generally defined using clinical signs, including arrested flatus/bowel movement, abdominal distension and vomiting and radiological findings, such as intestinal dilatation (23). The incidence of pre-operative bowel obstruction among the patients examined in the present study was 7.0%, a lower incidence than reported in previous studies. This finding may be attributed to the use of a more restrictive and objective definition of bowel obstruction; specifically, bowel obstruction was diagnosed if patients required emergency decompression via placement of a decompression tube or emergency surgery. This is a more objective means of assessment compared with the evaluation of clinical signs and radiological findings. Generally, patients with colon cancer who develop pre-operative bowel obstructions tend to experience distant metastasis. Although the mechanism by which metastasis develops is unclear (23), infiltration of micro tumor cells into the lymphatic vessels or veins and circulation due to increased intestinal pressure have been hypothesized.

CEA is a glycoprotein that was discovered by Gold and Freedman in 1965 and is present in the digestive tract of the fetus and the tumor tissue of endodermally-derived digestive organs (27). Although CEA was once considered to be a specific marker for gastrointestinal cancer, it is now recognized as a more general tumor marker. The pre-operative CEA level is now known to be associated with prognosis (7,21,28–30). Although a number of studies have established the cut-off value between a normal and high CEA level as 5 ng/ml (7,21,28), others have observed it to range between 10 ng/ml (29) and 15 ng/ml (30). In an experimental model, a CEA-producing tumor was identified to be more capable of liver metastasis than a non-CEA-producing tumor (31). Therefore, the higher the CEA level, the higher the malignant potential of the tumor. Patients with an elevated pre-operative CEA level may display micrometastases, particularly in the liver.

The 2006 ASCO Guidelines indicate that a pre-operative serum CEA level of >5 ng/ml is associated with a poor prognosis. However, this factor has not been adopted as an indicator for the assessment of indication for post-operative ACT due to insufficient data to support its use (32). Although the previous ESMO Clinical Practice Guidelines indicated a high serum CEA level as a high-risk factor for stage II colon cancer (33), the latest guidelines do not (4). As such, it remains controversial to consider a high CEA level as a high-risk factor or as an indicator for ACT. Nevertheless, using CEA levels offers the advantage of objectivity in an assessment, as an objective indicator is unlikely to be assessed differently by various interpreters.

Based on these previous findings and indications, we propose that stage II colon cancer patients who have undergone a curative resection should be considered to be at a high risk for recurrence if they present with a pre-operative CEA level of twice the cut-off value and/or with a pre-operative bowel obstruction. In the present study, patients in the low-risk group (n=291) who had presented with neither indicator were shown not have benefited from adjuvant therapy. In contrast, the patients in the high-risk group (n=86), who presented with one or both indicators and had undergone adjuvant therapy were shown to have experienced a significantly improved prognosis than those in the high-risk group who had not undergone adjuvant therapy; this was manifested by the 5-year recurrence-free rate of 94.4% observed in the ACT group compared with 71.1% identified in the AS group. In accordance with the proposal and results of the present study, patients who present with a pre-operative serum CEA level of twice the cut-off value or with a pre-operative bowel obstruction should be considered as candidates for adjuvant chemotherapy. Future studies into the prognostic risk factors for stage II colon cancer should examine the validity of this proposal by conducting a prospective investigation of the benefit of ACT for stage II colon cancer patients who present with the two proposed high-risk factors. Further research should also build on the findings using standard procedures at a single institution to obtain objective data to examine the correlation between prognosis and a variety of clinicopathological factors.

## Figures and Tables

**Table I:** Comparison of patient data by treatment group.

Variable	ACT group	SA group	P-value
Age, years (mean ± SD)	63.3±9.4	67.7±11.7	0.001^a^
Gender, n			
Male	104	141	0.744
Female	59	73	
Tumor location, n			
Left colon	96	129	0.832
Right colon	67	85	
Gross tumor type, n			
Invasive	19	20	0.498
Non-invasive	144	194	
Pre-operative CEA level, n			
≥NL	56	80	0.448
<NL	107	128	
Pre-operative CEA level, n			
≥NL×2	30	38	0.999
<NL×2	133	170	
Pre-operative CA19-9 level, n			
≥NL	12	20	0.690
<NL	55	73	
Tumor size, n			
≥Median	96	107	0.096
<Median	67	107	
Number of dissected LNs, n			
<12	9	31	0.006^a^
≥12	154	183	
Histology, n			
Others	19	18	0.301
Well/mod	144	196	
T factor			
T4	63	74	0.450
T3	100	140	
Adjacent organ invasion, n			
Positive	19	23	0.869
Negative	144	191	
Bowel obstruction, n			
Positive	10	15	0.836
Negative	153	199	
Lymphatic permeation, n			
Extensive	20	25	0.874
Slight	143	189	
Venous invasion, n			
Extensive	8	25	0.026^a^
Slight	155	189	

Significance was evaluated using the Fisher’s exact and Student’s t-tests;

a P<0.05.

ACT, adjuvant chemotherapy; CA19-9, carbohydrate antigen 19-9; CEA, carcinoembryonic antigen; LN, lymph node; mod, moderately-differentiated adenocarcinoma; NL, normal limit; SA group, surgery alone; well, well-differentiated adenocarcinoma.

**Table II:** Comparison of patient data by recurrence status.

Variable	Recurrent cases	Non-recurrent cases	P-value
Age, years (mean±SD)	66.0±11.8	65.8±10.8	0.927
Gender, n			
Male	34	211	0.420
Female	14	118	
Tumor location, n			
Left colon	33	192	0.208
Right colon	15	137	
Gross tumor-type, n			
Invasive	9	30	0.070
Non-invasive	39	299	
Pre-operative CEA level, n			
≥NL	25	111	0.024^a^
<NL	23	212	
Pre-operative CEA level, n			
≥NL×2	15	53	0.026^a^
<NL×2	33	270	
Pre-operative CA19-9 level, n			
≥NL	3	29	0.385
<NL	6	122	
Tumor size, n			
≥Median	24	179	0.643
<Median	24	150	
Number of dissected LNs, n			
<12	7	33	0.321
≥12	41	296	
Histology, n			
Others	5	32	0.799
Well/mod	43	297	
T factor, n			
T4	18	119	0.873
T3	30	210	
Adjacent organ invasion, n			
Positive	9	33	0.085
Negative	39	296	
Bowel obstruction, n			
Positive	6	19	0.112
Negative	42	310	
Lymphatic permeation, n			
Extensive	10	35	0.055
Slight	38	294	
Venous invasion, n			
Extensive	7	26	0.165
Slight	41	303	
Treatment, n			
SA	34	180	0.042^a^
ACT	14	149	

Significance was evaluated using the Fisher’s exact test and Student’s t-test.

a P<0.05.

ACT, adjuvant chemotherapy; CA19-9, carbohydrate antigen 19-9; CEA, carcinoembryonic antigen; LN, lymph node; mod, moderately-differentiated adenocarcinoma; NL, normal limit; SA, surgery alone; well, well-differentiated adenocarcinoma.

**Table III:** Univariate and multivariate analyses of the TTR of the SA group.

	Univariate analysis			Multivariate analysis		
		
Variable	HR	95% CI	P-value	HR	95% CI	P-value
Age (≥75 years vs. <75 years)	1.561	0.760–3.087	0.218			
Gender (male vs. female)	1.556	0.753–3.527	0.240			
Tumor location (left vs. right colon)	1.177	0.592–2.457	0.647			
Gross tumor-type (invasive vs. non-invasive)	2.518	0.941–5.684	0.064			
CEA (≥NL vs. <NL)	2.324	1.183–4.649	0.015^a^			
CEA (≥NL×2 vs. <NL×2)	3.353	1.513–7.165	0.004^a^	3.840	1.674–8.629	0.002^a^
CEA (≥NL×3 vs. <NL×3)	2.958	1.349–6.013	0.008^a^			
CA19-9 (≥NL vs. <NL)	1.828	0.262–8.496	0.492			
Tumor size (≥ median vs. <median)	0.765	0.383–1.503	0.437			
Number of dissected lymph nodes (<12 vs. >12)	1.898	0.760–4.132	0.158			
Histology (others vs. well/mod)	0.983	0.236–2.751	0.977			
T factor (T4 vs. T3)	1.059	0.496–2.129	0.876			
Bowel obstruction (yes vs. no)	3.482	1.301–7.859	0.016^a^	6.284	2.024–16.47	0.003^a^
Lymphatic permeation (extensive vs. slight)	2.720	1.150–5.748	0.025^a^	2.523	0.911–6.017	0.072
Venous invasion (extensive vs. slight)	1.654	0.562–3.923	0.328			

a P<0.05.

TTR, time to reccurrence; CA19-9, carbohydrate antigen 19-9; CEA, carcinoembryonic antigen; CI, confidence interval; HR, hazard ratio; mod, moderately-differentiated adenocarcinoma; NL, normal limit; SA, surgery alone; well, well-differentiated adenocarcinoma.

**Table IV:** Correlation between serum CEA levels of twice the cut-off value and recurrence.

	ACT group^a^		SA group^b^	
		
CEA	≥NL×2^c^	<NL×2	≥NL×2^c^	<NL×2
Recurrence				
Yes, n (%)	2 (6.7)	12 (9.0)	13 (34.2)	21 (12.4)
No, n (%)	28 (93.3)	121 (91.0)	25 (65.8)	149 (87.6)

Significance was evaluated using Fisher’s exact test;

a P=0.999,

b P=0.003,

c P=0.008.

ACT, adjuvant chemotherapy; CEA, carcinoembryonic antigen; NL, normal limit; SA group, surgery alone.

**Table V:** Correlation between bowel obstruction and recurrence.

	ACT group^a^		SA group^b^	
		
Bowel obstruction	Yes^c^	No	Yes^c^	No
Recurrence				
Yes, n (%)	0 (0.0)	14 (9.2)	6 (40.0)	28 (14.1)
No, n (%)	10 (100)	139 (90.8)	9 (60.0)	171 (85.9)

Significance was evaluated using Fisher’s exact test;

a P=0.999,

b P=0.018,

c P=0.051.

ACT, adjuvant chemotherapy; SA, surgery alone.
